# Gender distribution of adult patients on highly active antiretroviral therapy (HAART) in Southern Africa: a systematic review

**DOI:** 10.1186/1471-2458-7-63

**Published:** 2007-04-25

**Authors:** Adamson S Muula, Thabale J Ngulube, Seter Siziya, Cecilia M Makupe, Eric Umar, Hans Walter Prozesky, Charles S Wiysonge, Ronald H Mataya

**Affiliations:** 1 Department of Community Health, University of Malawi, College of Medicine, Chichiri, Blantyre, Malawi; 2 Department of Epidemiology, School of Public Health, University of North Carolina at Chapel Hill, North Carolina, USA; 3 Center for Health and Social Research, Woodlands, Lusaka, Zambia; 4 Department of Community Medicine, University of Zambia Medical School, Lusaka, Zambia; 5 Department of Population Studies, Chancellor College, University of Malawi, Zomba, Malawi; 6 Department of Community Health, University of Malawi,-College of Medicine, Chichiri, Blantyre, Malawi; 7 Division of Infectious Diseases, Department of Medicine, University of Stellenbosch, Tygerberg, South Africa; 8 UNAIDS, Geneva, Switzerland; 9 Department of Global Health, Loma Linda University School of Public Health, Loma Linda, California

## Abstract

**Background:**

HIV and AIDS are significant and growing public health concerns in southern Africa. The majority of countries in the region have national adult HIV prevalence estimates exceeding 10 percent. The increasing availability of highly active antiretroviral therapy (HAART) has potential to mitigate the situation. There is however concern that women may experience more barriers in accessing treatment programs than men.

**Methods:**

A systematic review of the literature was carried out to describe the gender distribution of patients accessing highly active antiretroviral therapy (HAART) in Southern Africa. Data on number of patients on treatment, their mean or median age and gender were obtained and compared across studies and reports.

**Results:**

The median or mean age of patients in the studies ranged from 33 to 39 years. While female to male HIV infection prevalence ratios in the southern African countries ranged from 1.2:1 to 1.6:1, female to male ratios on HAART ranged from 0.8: 1 to 2.3: 1. The majority of the reports had female: male ratio in treatment exceeding 1.6. Overall, there were more females on HAART than there were males and this was not solely explained by the higher HIV prevalence among females compared to males.

**Conclusion:**

In most Southern African countries, proportionally more females are on HIV antiretroviral treatment than men, even when the higher HIV infection prevalence in females is accounted for. There is need to identify the factors that are facilitating women's accessibility to HIV treatment. As more patients access HAART in the region, it will be important to continue assessing the gender distribution of patients on HAART.

## Background

There is increasing global interest to ensure that HIV infected persons have access to antiretroviral therapy. In the developed world, mortality from AIDS has significantly reduced, in part due to wide availability and accessibility to highly active antiretroviral therapy (HAART). In resource-limited countries however, although there has been steady progress in increasing accessibility to antiretroviral therapy (ART), most patients have no access to this life saving intervention [1]. Natrass suggests that the poor and vulnerable segments of society are likely to miss out from accessing HAART. In many societies, women are likely to be poor and socially vulnerable.

When patients in resource limited settings access HAART, treatment adherence has been reported to be comparable to industrialized countries [2]. The benefits of treatment have also been suggested to contribute to overall HIV prevention [3]. However the benefits of HAART in resource-limited countries may be restricted as even when patients receive HAART, they often do so very late in the disease [4].

Southern Africa is the region of the world that has been hardest hit by the HIV pandemic. For instance, Malawi's adult (15–49 years) HIV prevalence was estimated at 14.4% in the Malawi Demographic and Health Survey in 2004 [5]. Zambia's HIV prevalence in the 15–49 years age group was 15.6% in the Zambia Demographic and Health Survey 2000–01 [6]. In Namibia, 10% of the 15 to 49 years old were HIV infected in 2005 [7]. While most of the countries in the region have very high HIV prevalence estimates exceeding 10%, the Democratic Republic of Congo (DRC) has 4.5% of its population infected [8]. These figures from the DRC are likely to be unreliable as health infrastructure in the country outside the capital city is almost non-existent due to several years of civil war. Theobald et al have expressed concern that HAART scaling-up may "continue to serve the needs of dominant powerful groups in society and undermine a true public health approach" [9]. Women in most societies contribute the largest group of socially vulnerable members. There is therefore legitimate concern that women may not be accessing HAART programs as well as men due to personal and societal barriers. Krawczyk et al, have studied the barriers in accessing HIV care in the southern states of the United States [10]. Gender, sexual behaviors, stigma, lack of health insurance and poverty, and social support all had a bearing in influencing decisions to access care. In general, non-whites, women, and the poor are unlikely to access care, and when they do, it was usually late. In other studies however, men have been reported to seek care late at times when symptoms are far advanced [11].

The situation in southern Africa though has not been well described probably because comprehensive HIV care which incorporates HAART is a relatively new phenomenon. With the scaling up of HAART programs and comprehensive care going on in the continent, there is need to describe the distribution of patients obtaining HAART in terms of gender and age. Some of the questions that need to be addressed are: Is there gender balance in the distribution of patients obtaining HAART? What age groups are more likely to access HAART? Answers to these questions may help to guide researchers and policy makers to identify operational gaps, and suggest interventions that may improve accessibility of patients to health services. We aimed to obtain answers to these questions by conducting a systematic review of published literature following the suggestions of the Meta-analysis of Observations Studies in Epidemiology (MOOSE) Group published by Stroup et al [12].

## Methods

### Search Strategy and Selection Criteria

We searched for peer-reviewed articles and conference abstracts published in English language that reported on gender distribution of adult patients obtaining highly active antiretroviral therapy (HAART) in treatment programs in southern Africa. Different combinations of the following keywords: "antiretroviral", "anti-retroviral", "highly active antiretroviral therapy", "HAART", "AIDS treatment" and "HIV treatment" and a combination of these terms to each of the southern African countries were used. For the purposes of this paper, southern Africa was defined as all the 14 countries that make up the Southern African Development Community (SADC). These countries are: Angola, Botswana, Democratic Republic of Congo (DRC), Lesotho, Madagascar, Malawi, Mauritius, Mozambique, Namibia, Seychelles, South Africa, Swaziland, Tanzania, Zambia and Zimbabwe.

Electronic searches of MEDLINE, PsycINFO, African Journals Online (AJOL), Ingenta and Ovid electronic databases were done for reports published from 2000 to August 15, 2006. We also searched electronically for conference abstracts on Gateway [13] and manually for relevant HIV, Virology and retroviral conference abstracts. HIV prevention, treatment and support program reports from the Ministries of Health were obtained and reviewed manually. References from articles retrieved were examined to determine if they would lead us to other papers that had the information we were seeking.

We included a study or program document for review if it reported on the following within a treatment program: the gender distribution of patients on HAART, mean or median age of patients and time period of accumulation of patients on treatment of not less than 6 month. Journal articles and the other reports had to report on 'routine' program treatment and not randomized clinical trials or any aggregation of patients whose composition was determined by the authors or researchers i.e. we aimed to capture the natural flow of patients accessing HAART.

The following information was extracted from the studies and relevant program documents: authors, setting of study, duration of accumulation of cases included in the study, male and female distribution of cases, whether program required patients to pay for medication or medications were provided for free and mean or median age of patients on treatment.

We also contacted by email, 20 corresponding authors of the articles that we retrieved and HIV and AIDS national program officials asking them whether they were aware of any other reports on HAART provision in the southern African countries they were working.

In order to determine whether there was gender balance or imbalance among patients accessing HAART care, we compared the estimated gender ratio of HIV infection prevalence in a specific country to the gender ratio on treatment. We used the UNAIDS 2005 data of national HIV prevalence estimates [14]. This ratio was extrapolated and interpreted in such a way that we suggested that the same gender ratios would be expected to be obtained for patients accessing care. If the gender ratio between expected numbers of HIV infected and those receiving HAART care did not match, this was interpreted as evidence of inequity.

We did not attempt to assess whether any patients within a study area were included in only one study or report. As the purpose of the study was to assess the gender distribution of patients, patient serially recorded still served our purpose which was to estimate what was the gender distribution of patients at any particular time data were reported (multiple cross sectional studies).

## Results

We identified 21 reports that were eligible to be included in the review [15-35]. We obtained only 6 responses from the 20 emails that were sent to persons we had contacted. Table 1 reports on number of HIV infected adults distributed by gender and the gender ratios of estimated infections in southern African countries.

**Table 1:** Adult Male versus Female HIV prevalence distribution in Southern Africa, 2005 [14]

Country	Female Prevalence	Male Prevalence	Female: Male Ratio
Angola	160,000	120,000	1.3
Botswana	140,000	120,000	1.2
DRC	490,000	400,000	1.2
Lesotho	140,000	110,000	1.3
Madagascar	13,000	36,000	0.4
Malawi	480,000	370,000	1.3
Mozambique	920,000	680,000	1.4
Namibia	120,000	90,000	2.1
South Africa	2,900,000	2,400,000	1.2
Swaziland	120,000	90,000	1.3
United Republic of Tanzania	680,000	550,000	1.3
Zambia	540,000	460,000	1.2
Zimbabwe	930,000	570,000	1.6

Table 1 shows that South Africa has the largest number of HIV infected adults in Southern Africa. The gender ratios (female to male) of infected adults range from 0.4 in Madagascar to 1.6 in Zimbabwe. Four countries have 1.2 gender ratio, 6 have 1.3 and one each have 1.4 and 1.6. Table 2 below reports the number of patients on treatment and their demographic characteristics, including gender. Gender ratios on treatment have been computed and these are also presented in the table.

**Table 2:** Gender and Age Distribution of Patients on HAART in Selected Southern Africa

**Country**	**Authors**	**Period**	**Study Site**	**Number of patients on ARVs**	**Males (%)**	**Females (%)**	**Female :ratio**	**Mean/Median Age in Yrs**
Botswana	Ndwapi et al [15]	2002	Gaberone	306	135 (44%)	171 (56%)	1.3	36.8
	Wester et al [16]	1999–2004	Gaberone	153	62 (41%)	91 (59%)	1.5	36
	Bisson et al [17]	1999–2004	Gaberone	305	129 (42%)	175 (58%)	1.4	37
Malawi	Hosseinipour et al [18]	2003	Lilongwe	141	67 (48%)	74 (52%)	1.1	38.5
	van Oosterhaut et al [19]	2003	Blantyre	176	80 (40%)	96 (55)	1.2	39
	Zachariah et al [20]	2003–05	Thyolo	1634	572 (35%)	1062 (65)	1.9	33
	Ferradini et al [21]	2001–03	Chiradzulu	1308	481 (36%)	827 (64%)	1.7	34.9
	Hosseinipour et al [22]	2001–03	Lilongwe	625	376 (51.5%)	354 (48.5)	0.9	38
	Bonnet et al [23]	2001–03	Chiradzulu	1033	367 (35.5%)	666 (64.5%)	1.8	35
	Libamba et al [24]	2001–05	Malawi	13183	5274 (40%)	7909 (60%)	1.5	Not specified
	Phiri and Boxshall [25]	2001–05	Malawi	35841	14819 (35.6%)	23021 (64.4%)	1.6	33
	Malawi MoH [26]	2001–06	National	46,702	18213 (39%)	28,488(61%)	1.6	Not specified
Mozambique	Palombi et al [27]	2002–3	Machava	802	292 (36.4%)	510 (63.6%)	1.7	Not specified
South Africa	Stewart & oveday [28]	1999–04	National	8149	2824 (35%)	5325 (65%)	1.9	Not specified
	Coetzee et al [29]	2001–2	Khayelitsha	287	86 (30%)	201 (70%)	2.3	Not specified
	Hassan and Bosch [30]	2001–04	Eastern Cape	4147	1499 (36.1%)	2648 (63.9%)	1.8	Not specified
	Hassan and Bosch [30]	2001–05	Northern Cape	3156	1005 (31.8%)	2151 (68.2%)	1.5	Not specified
Swaziland	Ericsdotter [31]	2003–05	Mbabane	4800	2688 (56%)	2112 (44%)	0.8	Not specified
Tanzania	TACAIDS [32]	2004–6	National	12000	< 50%	> 50%	> 1	Not specified
Zambia	Stringer et al [33]	2004–05	Lusaka	21755	8109 (37%)	13646 (63%)	1.7	35
	Sinkala et al [34]	2004–05	Lusaka	22121	11691 (51.7%)	10691 (48.3%)	1.1	35
	Ministry of Health [35]	2002–05	National	3982	1638 (41%)	2344 (59%)	1.4	not specified

The reported median or mean age of patients reported ranged from 33 to 39 years. In 3 studies, there was a male predominance in treatment programs, one each from Malawi, Swaziland and Zambia. The majority of the studies however reported a > 1.0 female to male ratio. Ten studies reported a female to male ratio exceeding 1.6 (the highest ratio for HIV infected females to infected males reported in Table 1). All reports of programs enrolling > = 1000 patients reported female predominance.

## Discussion

Our study found that published data do not suggest that women are at a disadvantage when compared to men when accessing HAART in southern Africa. The predominance of women in HAART programs to an extent reflects the gender proportions of the adult population infected with HIV in southern Africa. For instance, the Zambia Demographic and Health Survey 2000–01 [6] national HIV prevalence for persons aged 15 yrs to 49 years was for 17.8% for females and 12.9% for males. As a proportion of all adults accessing HAART, women are in the majority outnumbering men.

Several authors have reported that women access HIV care earlier than men in the United States [36-38]. Others authors though have reported opposite findings [39]. The improvements in access of HIV testing among women attending antenatal care (ANC) has been reported as an important entry point for these women into HIV care and the support pipeline [40]. In southern Africa, there is also a drive to encourage women attending ANC to be tested for HIV in an effort to reduce vertical HIV transmission. Such drives could contribute to the higher access of women to HIV care services. The extent however to which HIV antenatal services have contributed to overall HAART programs need to be evaluated.

In some HIV testing programs outside of antenatal care, there has been a predominance of females over men in many settings [41]. In 2004 in Malawi, out of 283,467 persons tested for HIV across the country, 15% were women accessing antenatal care. However, in free standing testing services provided by the Malawi AIDS Counseling and testing Organization (MACRO), of the 33,441 people tested, only 31% were female [42]. This pattern of low female representation was observed at three sites. Whether women were under-represented because they had other access through antenatal care was not described. It has to be recognized also that although there may be more males accessing HIV testing, proportionally more women are likely to test positive than males in southern Africa. Women are the primary care givers in homes in virtually all countries in southern Africa and are likely to present to health facilities with sick family members. They also generally have greater interaction with health care services as they obtain maternity and family planning related care. Women's familiarity with health services may be working in their favour by facilitating their access to health services for HAART.

In many sub-Saharan countries, it is believed that women are mainly infected with HIV by their own spouses [43,44]. It is therefore possible that men may be reluctant to acknowledge that they may be infected compared to women who may be forthright in accessing care believing, and little stigmatized as HIV is perceived to have been acquired from a spouse. Men on the other hand may fear that they may be perceived to have acquired infection outside of marriage. Krawczyk et al reported that anticipated stigma was associated with delay in seeking HIV care in the United States [10].

Bachmann has reported that late initiation of HIV treatment was less cost-effective than early initiation of therapy in southern Africa [45]. It is therefore pertinent that an assessment is made to determine at which points in the care continuum delays occur in accessing care. Delays can occur due to patient factors and intrinsic health system factors [46].

Badri et al have suggested that for resource-limited settings, prioritizing symptomatically very sick patients for HAART is likely to be the most rational way of scaling-up HAART programs [47]. Palombi et al however argued that "this minimalistic approach" is likely to put many people in harms way unnecessarily by delaying therapy to a point where it may not be as effective anymore due to unredeemable immune compromise [48]. The harm that may result from delay in initiating therapy has probably been shown in Malawi by Zachariah et al [49] where advanced HIV disease is shown to significantly contribute to mortality among patients on HAART. Hosseinipour et al [50] have also demonstrated a high mortality of patients within the first few weeks into entry to HAART programs. If males are presenting late into treatment programs they are likely to suffer significant mortality and therefore limit the benefits of HAART programs. There is need to assess whether males have worse mortality while on HAART compared to females. A study by Mshana et al reported that barriers in accessing HAART included difficulties in identifying a treatment buddy, transport costs to treatment centers, fear of disclosure of HIV status and perception that hospitals were unfriendly and confusing [51]. The treatment "buddy approach" is aimed to enhance adherence to therapy as patients have an identified treatment support person. However, as the requirement to identify a treatment buddy seems widespread in southern Africa [51],[52] there is need to assess its pros and cons. For instance, could it be that men have trouble identifying treatment buddies and so may be under-represented in treatment programs? Any suggestion to change the policy should also consider the potential loss in patient treatment adherence that may occur in the absence of a treatment buddy, who is currently perceived as key in supporting patients.

HIV prevalence is highest in the 30–34 years group at 19.2% and 29.4% respectively. Of particular note also is the fact that the mean or median ages for patients on HAART treatment ranged from 33 and 38 years. This suggests that the groups with highest likelihood of symptoms i.e. approximately 10 years following infection are also being fairly represented in treatment programs. It is also important to note that most recently infected person do not become clinically eligible for HAART under the current guidelines in operation in most of the countries. People who are starting treatment may have acquired infection 5 to 10 years before and only becoming eligible for HAART due to waning immunity. As this study was restricted to adult treatment programs, the situation in children may be different.

With reference to Table 2, it is interesting to note that in general the gender distribution seem to be maintained across programs despite of differences as to whether patients were required to pay for medications or not.

It is interesting to note that the study by Hosseinipour et al [18] reported on a period when a fee-paying HAART treatment program was running in Lilongwe i.e. before free treatment was started. During this period, there were slightly more males (51.5%) than females (48.5%) and patient drop out was at 35.5%. In Zambia high drop-out rate were also observed prior to 2004 when most of the patients were obtaining HAART on fee-paying basis [28]. At this time, there were 52.1% females versus 47.9% males on HAART. Subsequent to the introduction of national free HAART services, there are more women than males on treatment. However, in general there was still female gender predominance even in paying treatment programs.

There are several limitations of this review. Firstly data were mostly obtained from peer reviewed journals indexed in the databases that we reviewed. Treatment programs that did not publish reports were therefore missed. We however complemented the lack of peer-reviewed publications with abstracts from conferences. Again this source of data may be selective. If HAART treatment that did not publish their experiences have different gender distribution from those that published, then our conclusions may have to be revised. We however do not believe that the non-published experiences were overall systematically different from the reports reviewed in our study. The strength of our study also comes from the fact that we have presented data obtained at different time periods within the same country but still consistently showed that the gender distribution was in favour of females. This probably offers strongest evidence that the gender pattern is unlikely to be spurious or fluctuating but rather it is a stable phenomenon. The report from Tanzania [32] was less informative as the actual numbers were not reported. The percentages of patients on HAART were however provided.

As our search was limited to publications in English, it was possible that we missed some relevant articles published in French (Democratic Republic of Congo) and Portuguese (Angola and Mozambique). We were however able to find articles in English from Mozambique, a Portuguese-speaking country, and these were included in the review. There was also likely to be overlapping of patients cohorts among the reports. For instance, in any country where both national treatment figures and localized patient numbers where obtained, many of the patients at the local setting would also be counted within the national figures. We do believe that such double counting in fact emphasizes the point that even when both larger and smaller patient cohorts were assessed, the gender distributions were maintained.

## Conclusion

The available published data suggest that females may not be disadvantaged in accessing highly active anti-retroviral therapy in Africa. From the limited available reports however, there was either gender balance within treatment programs or predominance of females within treatment programs. There is need to identify facilitating factors that enhance high uptake of women on HAART programs in order to sustain the current successes. There is also need to identify barriers against accessing care by males. This will ensure that both genders have fair access to treatment programs.

## Abbreviations

ART: antiretroviral therapy

ARVs: antiretrovirals

DRC: Democratic Republic of Congo (ex Zaire)

HAART: highly active antiretroviral therapy

HIV: Human immunodeficiency virus

MACRO: Malawi AIDS Counseling and Testing Organisation

MOH: Ministry of Health

MOOSE: Meta-analysis of Observations Studies in Epidemiology

SADC: Southern African Development Community

## Competing interests

The author(s) declare that they have no competing interests.

## Authors' contributions

ASM conceived the idea, collected data, participated in analysis and drafting of manuscript.

TJN collected data, participated in analysis and drafting of manuscript.

SS collected data, participated in analysis and interpretation of findings and drafting of manuscript.

EU participated in interpretation of data and the drafting of manuscript

CMM participated in interpretation of data and the drafting of manuscript.

HWP collected data, contributed to drafting manuscript and interpretation of data.

CSW collected data, participated in interpretation and writing of manuscript.

RHM: collected data, participated in analysis and drafting of manuscript.

## Pre-publication history

The pre-publication history for this paper can be accessed here:


